# Supplementary material for: Conserved long-range base pairings are associated with pre-mRNA processing of human genes
Source: Nat Commun. 2021 Apr 16;12:2300. doi: 10.1038/s41467-021-22549-7 (PMC8052449; doi:10.1038/s41467-021-22549-7)
Supplement: Supplementary file 1 — Supplementary Information [file 41467_2021_22549_MOESM1_ESM.pdf]

SUPPLEMENTARY INFORMATION

Conserved long-range base pairings are associated with pre-mRNA processing of human genes

Svetlana Kalmykova[1], Marina Kalinina[1], Stepan Denisov[1], Alexey Mironov[1], Dmitry Skvortsov[2], Roderic Guigó[3], and Dmitri Pervouchine[1,*]

[1]Skolkovo Institute of Science and Technology, Moscow 143026, Russia

[2]Faculty of Chemistry, Moscow State University, Moscow 119234, Russia

[3]Center for Genomic Regulation and UPF, Barcelona 08003, Spain

[*]e-mail: d.pervouchine@skoltech.ru

List of Figures

S1 PCCR energy and length 5
S2 Bona fide long-range RNA structures 6
S3 Properties of PCCRs 7
S4 Enrichment of GO terms 8
S5 E-values 9
S6 Structural alignments for QRICH2, MRPL42, and EREG 10
S7 Conservation-derived scores 11
S8 Distance to intron ends by random shifts 12
S9 CAGE-tags and poly(A)-seq clusters 13
S10 Matched Intron Lengths 14
S11 Mutations 15
S12 RBPs depleted in CCRs 16
S13 MFE correlation 17
S14 Transcript starts and ends inside PCCR 18

List of Tables

S1 *Bona fide* RNA structures not satisfying search criteria 19
S2 Time and accuracy of PrePH 20
S3 Accession numbers of RBP shRNA-KD 27
S4 Accession numbers of RBP eCLIPs 30

Supplementary Methods

PrePH

The algorithm of PrePH (the PREdiction of PanHandles), which borrowed some essential ideas from its predecessor IRBIS (*1*), and can be considered as a sparse implementation of Smith-Waterman algorithm for local sequence alignment (*2*) with specific enhancements that look for complementarity of k-mers. The main feature that enables efficient computations on the genome-wide scale is that PrePH scoring scheme is based on k-mers instead of single nucleotides, i.e., we disregard structural units that contain less than k base-paired nucleotides in a row. In order to compute the free energy contribution, we consider only a limited set of structural elements (stacked base pairs, bulges, and interior loops), neglect contributions from dangling ends and terminal mismatches, and disregard branching structures. Given a pair of sequences of lengths M and N, we sequentially compute three dynamic programming matrices D_{ij}, B_{ij}, and S_{ij} of size $M \times N$ as follows. We denote by D_{ij} the minimum energy (the maximum score) of the interaction that ends at the position i in the first sequence and the position j in the second sequence. We denote by B_{ij} the matrix that stores start coordinates of the base pairing that ends at the positions i and j, while the matrix S_{ij} stores the structure of the interaction in dot-bracket notation. The matrices B_{ij} and S_{ij} are needed for backtracking, making it unnecessary to restore the path of the alignment. To lower the memory usage from O($M \times N$), we split the algorithm into four main steps. In the preliminary step, we precomputed the hybridization energy for all possible pairs of ideally complementary k-mers based on the table of stacking energies obtained from (*3*). As a result, we obtain a $4^k \times 4^k$ matrix, whose elements are the free energies of the interaction of each k-mer with its ideal reverse complement. The pairs of k-mers that are not ideally complementary are given $+\inf$ energy; however, we also allow a small number of GT base pairs by listing all possible pairs of a given k-mer with the number of GT base pairs not greater than the given threshold (typically, two). The pre-computation of pairing energies for k-mers is performed only once and can be used several times afterwards. Given two input sequences, the algorithm extracts k-mers by and creates an index table. In the second step, PrePH scans for possible interaction sites. It looks up the matrix of precomputed k-mer stacking energies for each k-mer index in the first sequence and each k-mer index in the second sequence. All entries that were ideally complimentary on the first step are stored in D, B, and S matrices. While all k-mers that are not complementary and therefore should not start an alignment are set to $+\inf$. On the third step, which implements a modified local alignment algorithm, we

iterate through the sequences and elongate the stacking region in 9 different directions: one more pair of stacking nts, loop11, bulge10, bulge01, bulge20, bulge02, loop21, loop12, loop22 if we find non-zero energy value, i.e.

$$E' = D[i][j] + \min \begin{cases} stackingE[i-1][j+1][j][i] \\ bulge1E + stackingE[i-1][j+2][i-1][j+1][j][i] + kSE[J-1-k][I+k] \\ bulge1E + stackingE[i-2][j+1][j][i] + kSE[J-k][I+1+k] \\ bulge2E + TermAU([i-1][j+3],[j][i]) + kSE[J-2-k][I+k] \\ bulge2E + TermAU([i-3][j+1],[j][i]) + kSE[J-k][I+2+k] \\ loop11E[i-2][j+2][j][i][i-1][j+1] + kSE[J-1-k][I+1+k] \\ loop12E[i-2][j+3][j][i][i-1][j+1][j+2] + kSE[J-2-k][I+1+k] \\ loop12E[j][i][i-3][j+2][j+1][i-2][i-1] + kSE[J-1-k][I+2+k] \\ loop22E[i-3][j+3][j][i][i-2][i-1][j+1][j+2] + kSE[J-2-k][I+2+k] \end{cases},$$

where E' is the energy to be calculated; kSE is the precomputed k-mer stacking energy matrix; $stackingE$ is the matrix of stacking energies; $bulge1E$ is the energy of a 1-nt bulge; $bulge2E$ is energy of a 2-nt bulge; $TermAU$ is a function that penalizes for AU (or GU) terminating helix; $loop11E$ is the matrix of 1×1 loop energies; $loop12E$ is the matrix of 1×2 loops energies; $loop21E$ is the matrix of 2×1 loop energies; $loop22E$ is the matrix of 2×2 loop energies; $I = M - k - i$; $J = N - j - 1$.

The calculation of the energy is based on the ViennaRNA package algorithm for interior loops (*3*) with some modifications. The energy parameters are derived from the Turner energy model (*4*). Stem energies are calculated as the base pairs stacking energy of two consecutive nucleotides. The energies of bulges and interior loops usually contain a length-dependent term. Additionally, we allow for a loop structure or a bulge structure only if k preceding and succeeding nucleotides are complementary. This is implemented as an additional term in the equation that extracts k-mer stacking energy from the pre-computed matrix. The algorithm compares the obtained new energy with global minimal energy and updates it if necessary. It also stores the start and the end coordinates of the most energy-efficient path.

In this implementation, PrePH needs only marginally more time to identify suboptimal interactions on the fourth step. We limit our search to the suboptimal structures that don't intersect. Two structures are called non-intersecting if the complementary regions of both sequences of one structure do not have nucleotides in common with complementary regions of both sequences of the second structure (it is still allowed to have intersecting regions in only one of the two sequences). To obtain the suboptimal structures, the program sets to zeros the rectangle corresponding to the optimal alignment that was already found, looks for new minimal energy in the D_{ij} matrix, and checks whether the corresponding alignment intersects the optimal one. If it does, the energies along this path are set to zero. If not, the alignment is returned. These steps are repeated until all the energies below the energy threshold are considered.

The program returns a table of unique identifiers for every predicted RNA structure, its coordinates in the

genome, energy in kcal/mol, and structure in dot-bracket notation. The whole pipeline is implemented as a series of python scripts and allows parallel implementation.

Supplementary Figures

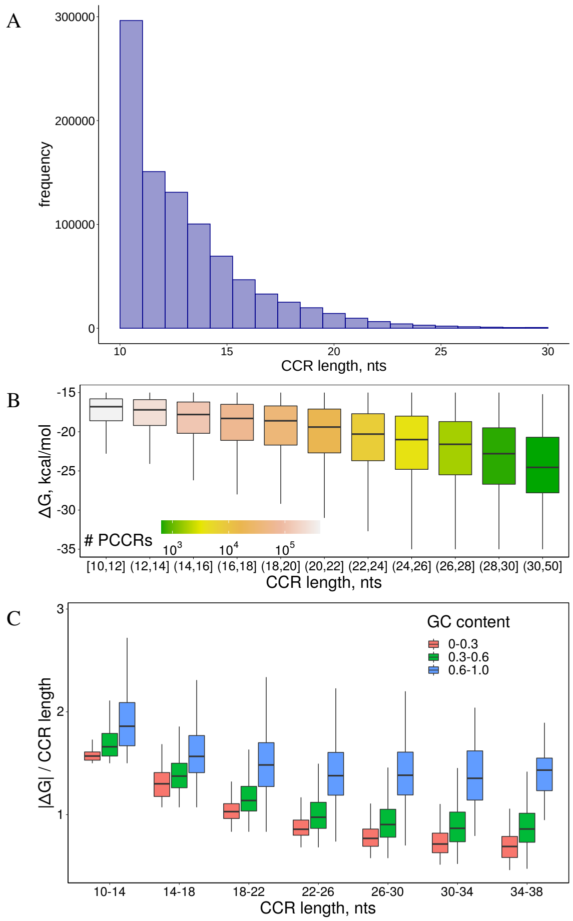

Figure S1: PCCR energy and length. **(A)** The distribution of CCR lengths. **(B)** The distribution of PCCR energies in different length groups. Colors represent sample sizes (n), i.e., the number of PCCRs in each length group. **(C)** The density of PCCR energy (ΔG per CCR length) as a function of GC content. Sample sizes in the $[0, 0.3]$ group are 14555, 42080, 34552, 13832, 3394, 922, and 179, respectively (left to right). Sample sizes in the $(0.3, 0.6]$ group are 95508, 332721, 119836, 31991, 6436, 1300, and 268, respectively (left to right). Sample sizes in the $(0.6, 1.0]$ group are 37620, 156141, 19749, 4182, 754, 142, and 24, respectively (left to right). In all panels, boxplots are represented by the median, upper and lower quartiles, upper and lower fences; outliers not shown.

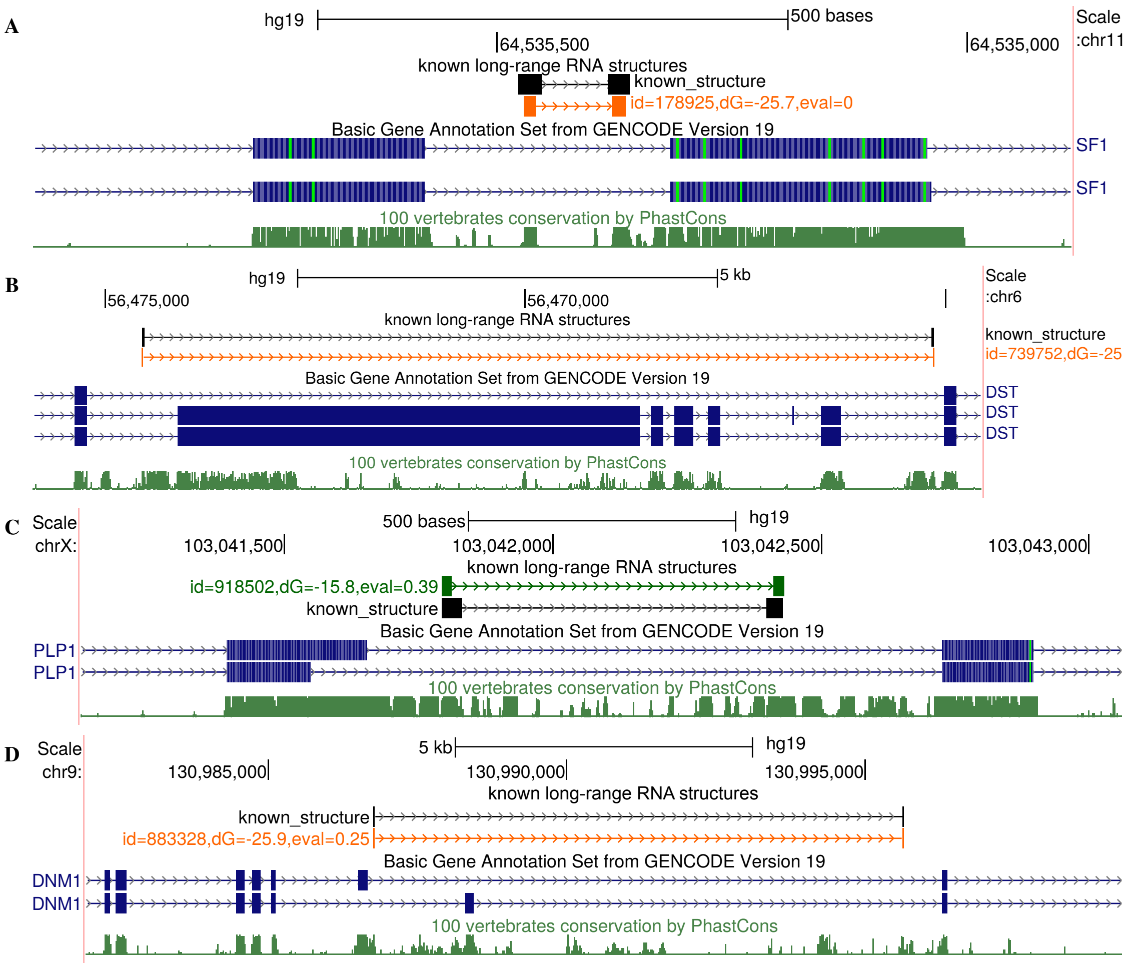

Figure S2: Long-range RNA structures in *SF1* (A), *DST* (B), *PLP1* (C), and *DNM1* (D) from earlier reports (Table 1). The structure in *ENAH* gene is shown separately in Figure 6A

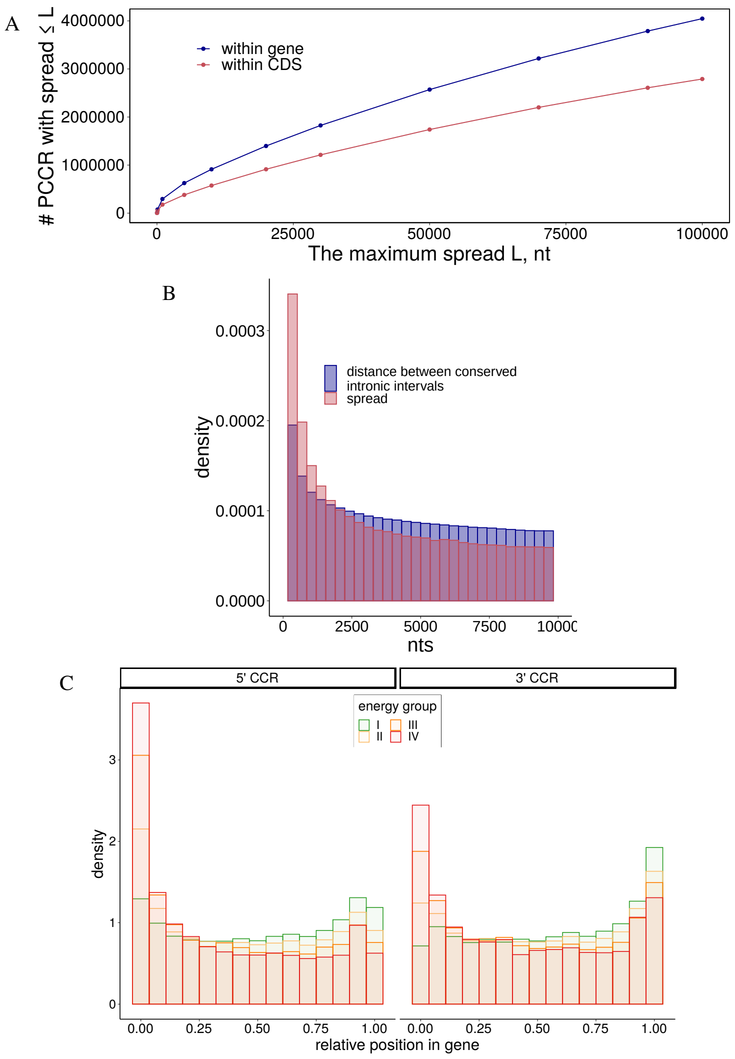

Figure S3: Properties of PCCRs. **(A)** The number of PCCRs as a function of maximum distance between CIR (L) in whole genes and within protein-coding parts. **(B)** The distribution of spreads and the distribution of pairwise distances between CIR. **(C)** The distribution of CCR positions along the gene.

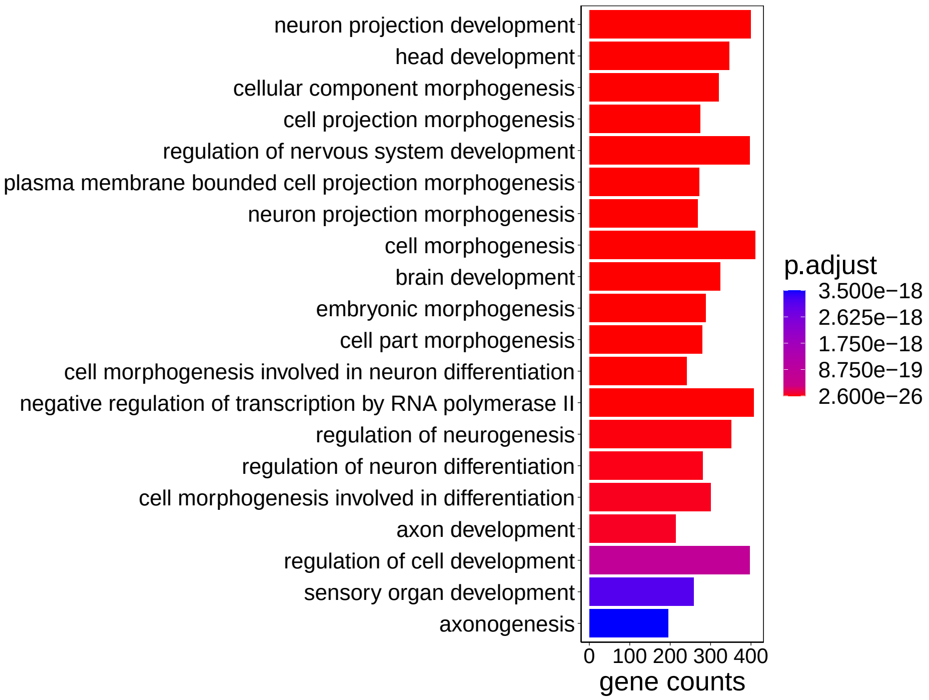

Figure S4: Enrichment of GO terms in genes with PCCRs as compared to genes matched by length, but without PCCRs.

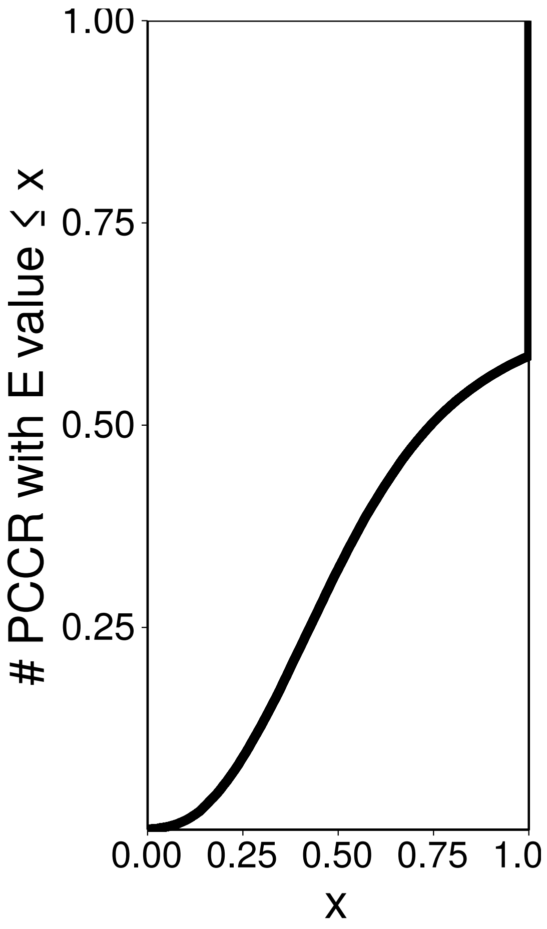

Figure S5: Cumulative distribution of E-values.

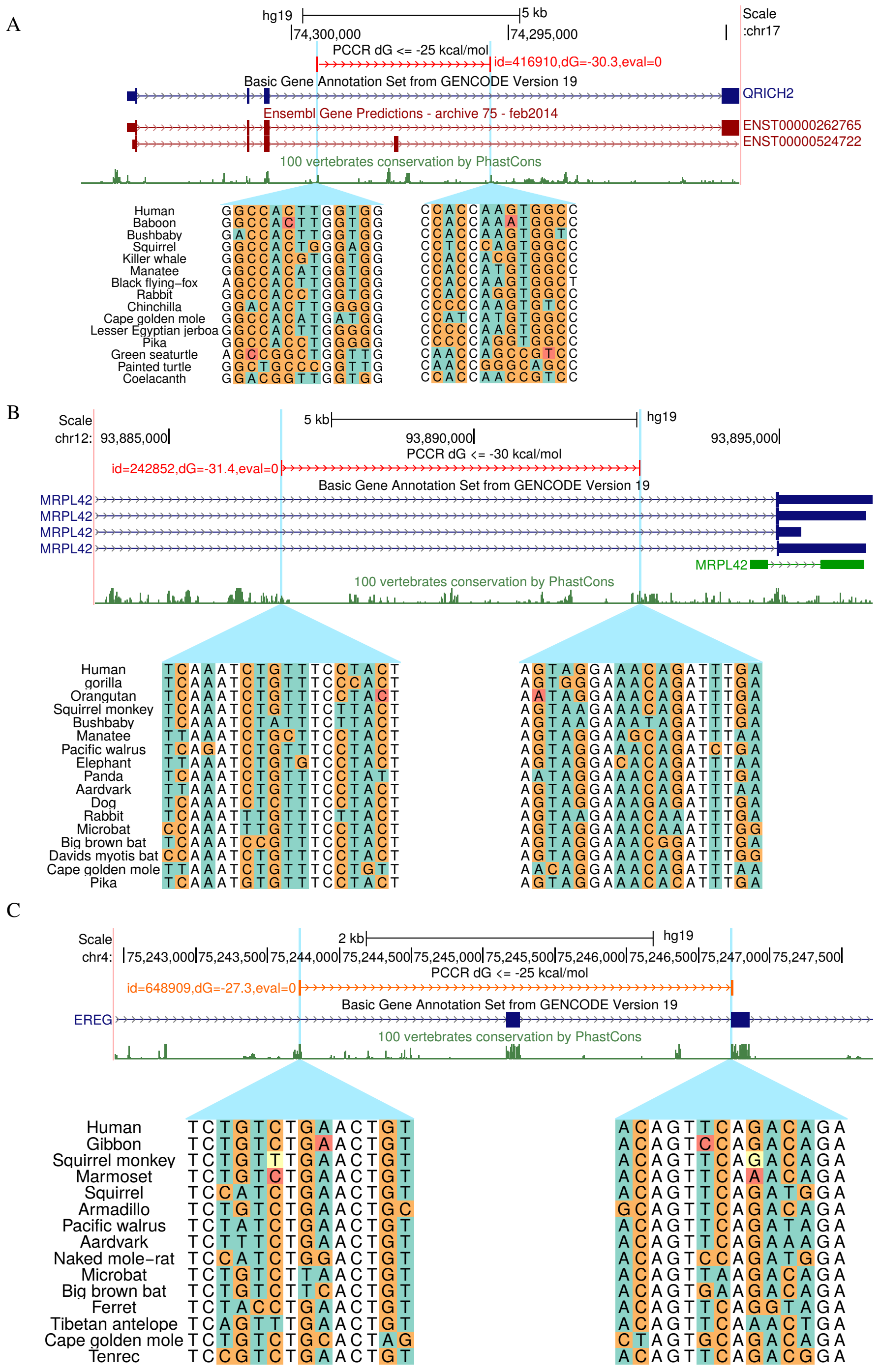

Figure S6: Structural alignments for PCCRs in the Glutamine-rich protein 2 gene (A), the 28S ribosomal protein L42 gene (B), and Epiregulin (C).

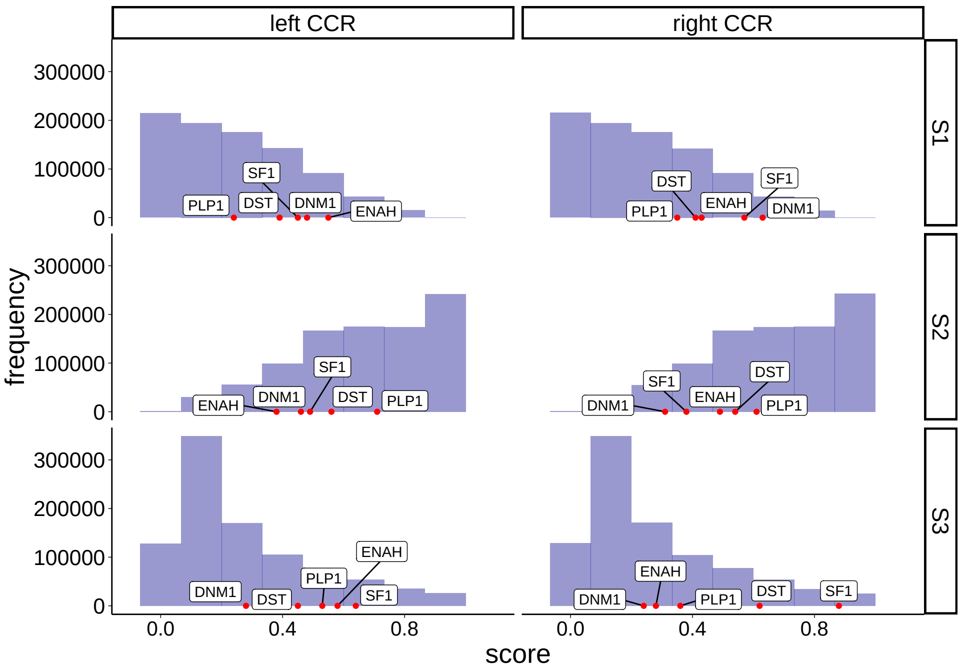

Figure S7: Conservation-derived scores. s_1 is the difference between the average phastCons scores within CCR and within 300 nt flanking regions (the larger, the more significant). s_2 is the average phastCons score within CCR and 300 nt around it (the smaller, the more significant). s_3 is the length of a CCR relative to the length of its parent CIR (the larger, the more significant). The respective values for *bona fide* RNA structures are shown by red dots.

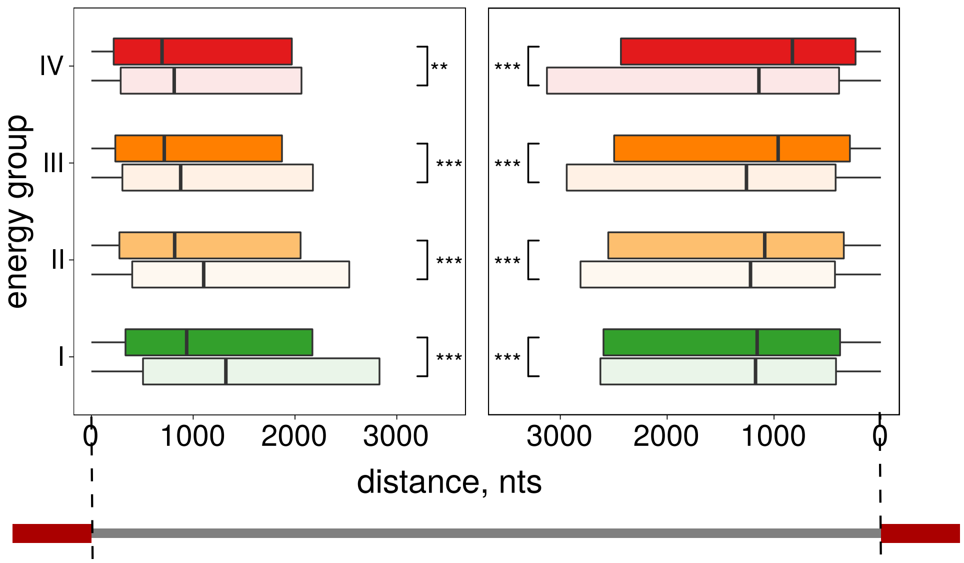

Figure S8: CCRs are located on average closer to intron ends than intervals obtained by random shift. Dark colors correspond to energy groups in Figure 3. Bright colors correspond to the respective random shift controls. In all panels, boxplots (represented by the median, upper and lower quartiles, upper and lower fences; outliers not shown) correspond to $n = 40$ randomizations; *, **, and *** denote a statistically discernible difference at the 5%, 1%, and 0.1% significance level for a two-tailed Mann-Whitney test.

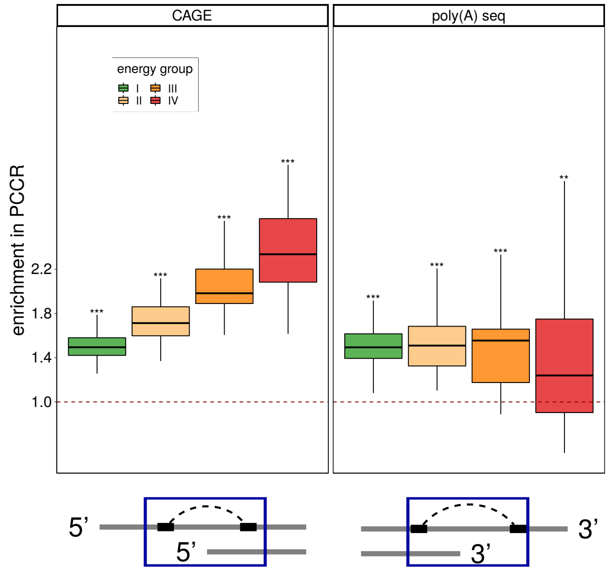

Figure S9: Expressed CAGE-tags and poly(A)-seq clusters are enriched within PCCRs. In all panels, boxplots (represented by the median, upper and lower quartiles, upper and lower fences; outliers not shown) correspond to $n = 40$ randomizations; *, **, and *** denote a statistically discernible difference at the 5%, 1%, and 0.1% significance level for a two-tailed Wilcoxon test with H_0 : enrichment $= 1$.

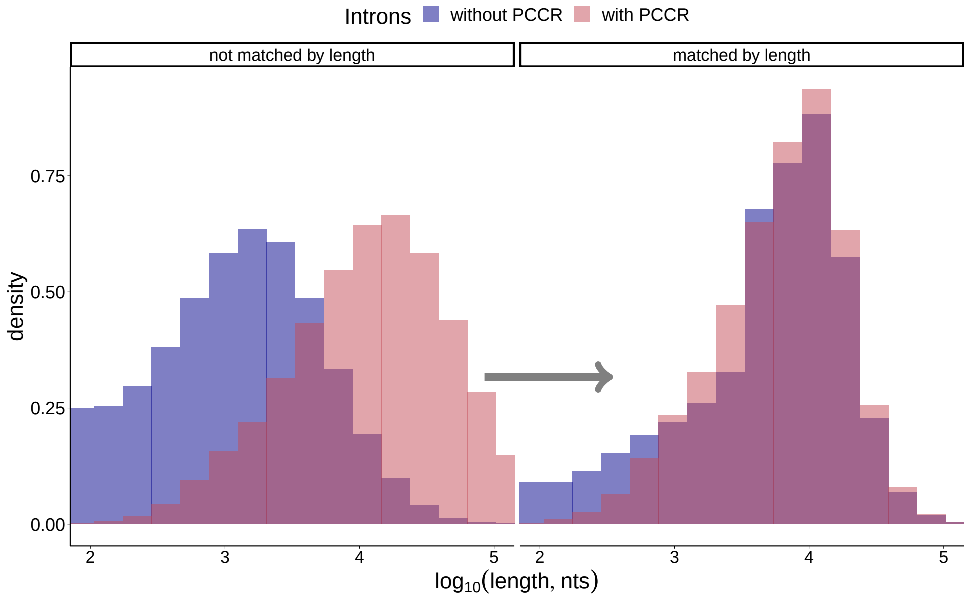

Figure S10: Matched Intron Lengths

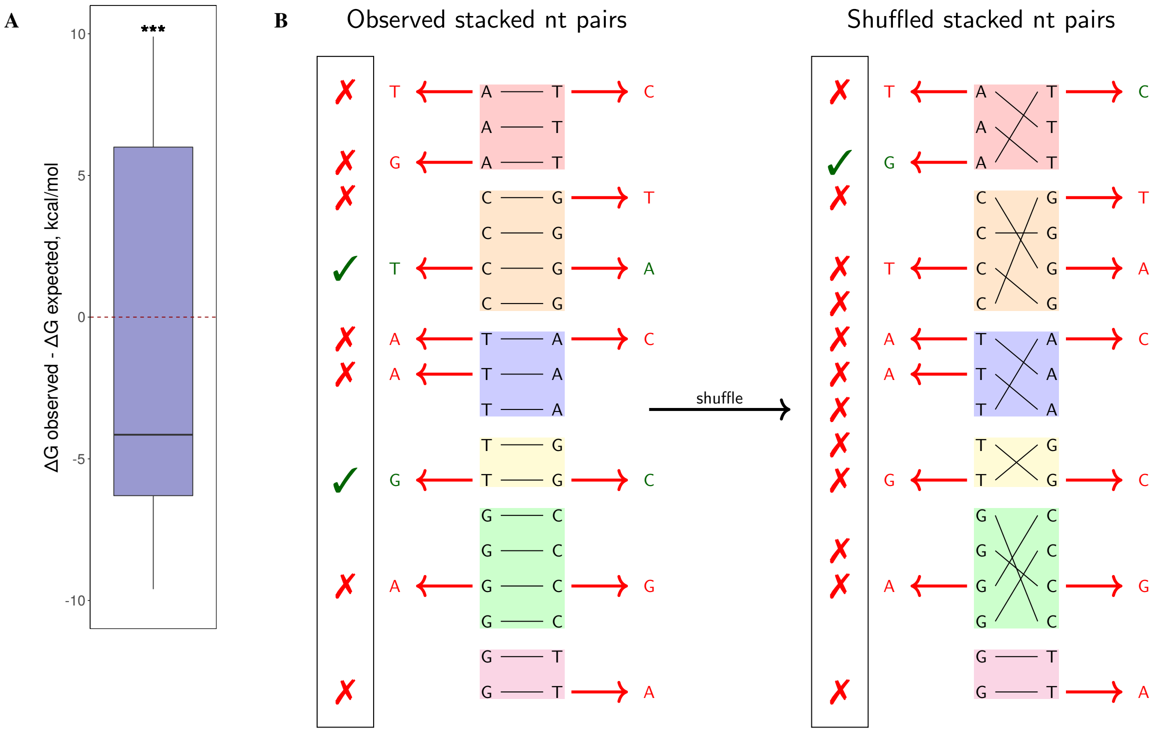

Figure S11: Mutations. **(A)** Change of ΔG in PCCRs with compensatory mutations ($n = 21920$). The boxplot is represented by the median, upper and lower quartiles, upper and lower fences; outliers are not shown; *** denotes a statistically discernible difference at the 0.1% significance level for a two-tailed Wilcoxon test with respect to $H_0 : \Delta G\text{observed} - \Delta G\text{expected} = 0$. **(B)** Estimation of enrichment with compensatory mutations.

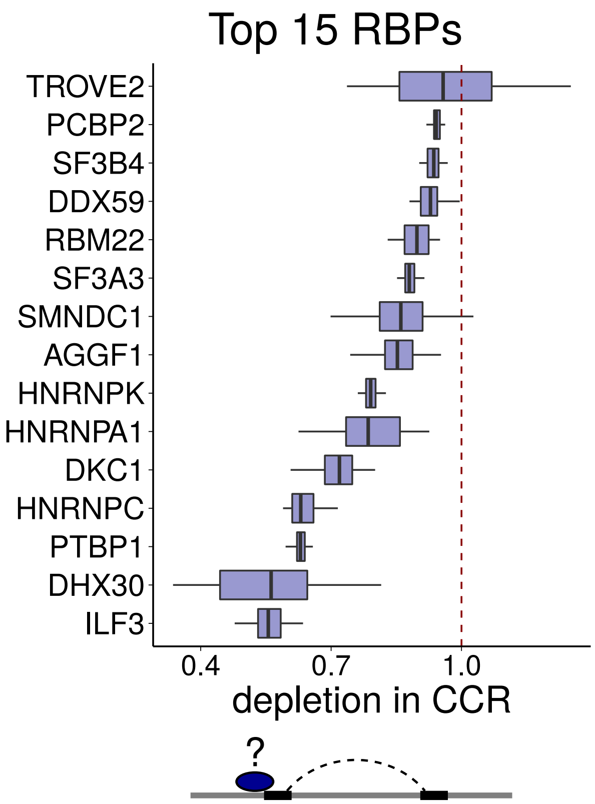

Figure S12: Top 15 RBPs with the most depleted binding within CCRs. Boxplots (represented by the median, upper and lower quartiles, upper and lower fences; outliers not shown) correspond to $n = 40$ random shifts of CCR within CIR.

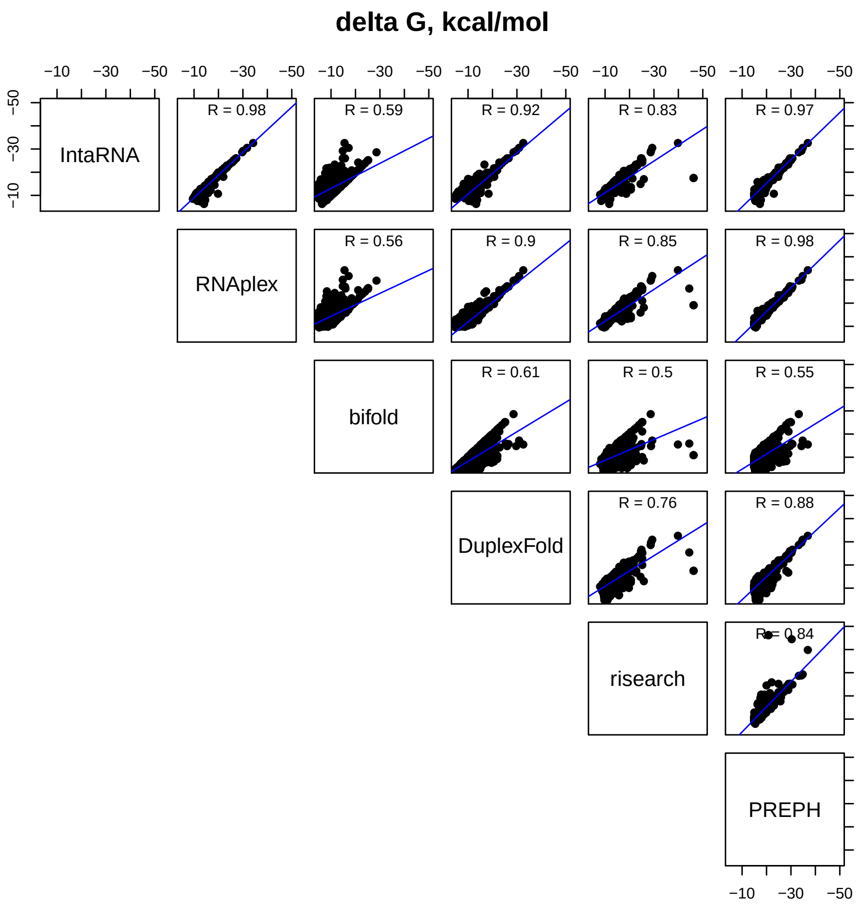

Figure S13: Correlation of predicted minimum free energies (MFE).

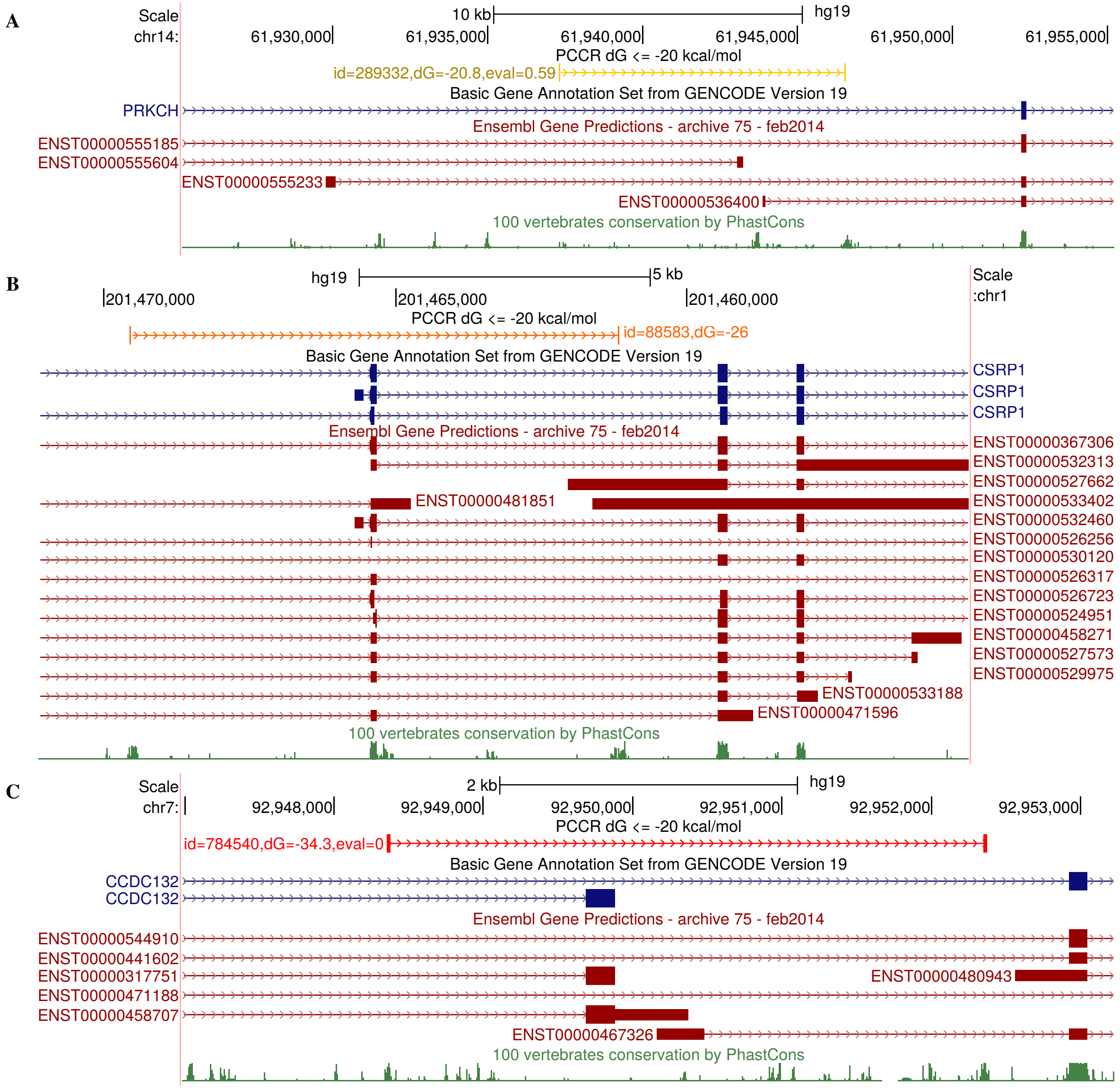

Figure S14: Transcript starts and ends inside PCCRs in *PRKCH* (A), *CSRP1* (B), and *CCDC132* (C) genes.

Supplementary Tables

Gene	Location	Approx. coordinates	Reason not found	References
SMN2, SMN1	exon 7	chr5:70,247,788-70,248,263	only 8-nt-long	(*5–8*)
TERT	exons 7 and 8	chr5:1,271,103-1,277,432	CCR in repeats	(*9*)
NEAT1	hNEAT1-S	chr11:65,168,509-65,233,788	lncRNA	(*10*)
MAPT (tau)	exon 10	chr17:44,087,754-44,087,801	only 6-nt-long	(*11–14*)
FGFR2	stem after exon IIIb	chr10:123,277,117-123,278,208	$\Delta G = -14.6$ kcal/mol	(*15*)
TNNT2 (cTNT)	exon 5	chr1:201,338,988-201,339,019	only 8-nt-long and no k=5	(*16*)
FTL	iron RE	chr19:49,468,567-49,468,639	outside CIR	(*17*)
CFTR	exon 11	chr7:117,199,585-117,199,685	lies in exon	(*18*)
GH1 (HGH)	stem before exon 3	chr17:61,995,469-61,995,577	multiple bulges in a row	(*19*)
PSEN2	stem-loop in exon 5	chr1:227,073,272-227,073,294	lies in exon, too short	(*20*)
FN1	stem-loop in EDA exon	chr2:216,245,605-216,245,671	lies in exon, too short	(*21*)
COL2A1	stem-loop following a weak 5'-ss	chr12:48,393,652-48,393,708	$\Delta G = -13.9$ kcal/mol	(*22*)
ATE1	exon 7b	chr10:123,631,345-123,659,239	$d = 27,856 > 10,000$	(*23*)

Table S1: Experimentally-validated (*bona fide*) RNA structures in human genes that didn't satisfy search criteria. Gene name, location within the gene, and approximate coordinates in GRCh37 human genome assembly are listed.

PREPH	RNAplex	DuplexFold	risearch	IntaRNA	bifold
191.4	218.2	234.2	279.6	449.5	1301.2

(A)

	PREPH	IntaRNA	RNAplex	risearch	DuplexFold	bifold
PREPH	1	0.81	0.85	0.8	0.84	0.61
IntaRNA	0.67	1	0.86	0.76	0.72	0.56
RNAplex	0.72	0.86	1	0.8	0.77	0.5
risearch	0.68	0.76	0.8	1	0.67	0.48
DuplexFold	0.79	0.8	0.85	0.74	1	0.57
bifold	0.78	0.83	0.78	0.73	0.77	1

(B)

Table S2: Time and accuracy of PrePH with respect to IntaRNA2.0, RIsearch2, RNAplex, DuplexFold, and bifold. **(A)** The run time (sec) on 1000 pairs of randomly chosen conserved intronic sequences from the human genome. **(B)** The number of base pairs that were common between PrePH and each of the other programs as a fraction of the number of base pairs predicted by PrePH alone.

Target	KD1	KD2	Ctrl1	Ctrl2
AARS	ENCFF359DJN	ENCFF809IPD	ENCFF572NPL	ENCFF689YCR
AATF	ENCFF283ZEU	ENCFF510KSY	ENCFF057JNE	ENCFF294SKR
ABCF1	ENCFF512RCL	ENCFF375JLH	ENCFF236XCF	ENCFF901WHO
ACO1	ENCFF493XEK	ENCFF974YWT	ENCFF566XUG	ENCFF486GXG
ADAR	ENCFF437CZI	ENCFF827TVS	ENCFF784SML	ENCFF487UNM
AGO1	ENCFF888QAR	ENCFF018TKU	ENCFF057JNE	ENCFF294SKR
AKAP1	ENCFF446GNQ	ENCFF554EIV	ENCFF989TXQ	ENCFF558ZFO
AKAP8	ENCFF718DSO	ENCFF762NBR	ENCFF093UUE	ENCFF870WUB
AKAP8L	ENCFF963ZJZ	ENCFF859HYF	ENCFF566XUG	ENCFF486GXG
APOBEC3C	ENCFF833UXW	ENCFF360ASR	ENCFF869IKC	ENCFF996UTY
ASCC1	ENCFF037MOS	ENCFF948YWB	ENCFF989TXQ	ENCFF558ZFO
ATP5C1	ENCFF924MNW	ENCFF120KHG	ENCFF236XCF	ENCFF901WHO
AUH	ENCFF447RFE	ENCFF326XLG	ENCFF742NXL	ENCFF340UAV
BCCIP	ENCFF085YWZ	ENCFF813CJQ	ENCFF572NPL	ENCFF689YCR
BCLAF1	ENCFF878AAZ	ENCFF587FCY	ENCFF572NPL	ENCFF689YCR
BOP1	ENCFF380PZQ	ENCFF570FGE	ENCFF738YFT	ENCFF293RIK
BUD13	ENCFF944UDR	ENCFF526PAV	ENCFF236XCF	ENCFF901WHO
CALR	ENCFF564GBC	ENCFF862YLD	ENCFF433MPP	ENCFF650KXR
CCAR1	ENCFF874TAD	ENCFF981UXB	ENCFF930OYV	ENCFF934UWJ
CCAR2	ENCFF867UDD	ENCFF734UFY	ENCFF261BLR	ENCFF966QBV
CEBPZ	ENCFF347KWA	ENCFF022CJU	ENCFF236XCF	ENCFF901WHO
CELF1	ENCFF529SHX	ENCFF965ZBQ	ENCFF566IXO	ENCFF394YXV
CIRBP	ENCFF719TWR	ENCFF270FIC	ENCFF989TXQ	ENCFF558ZFO
CKAP4	ENCFF348ABY	ENCFF855TVS	ENCFF566XUG	ENCFF486GXG
CNOT7	ENCFF784QPD	ENCFF711NKA	ENCFF869IKC	ENCFF996UTY
CPSF6	ENCFF183DNU	ENCFF657FCV	ENCFF738YFT	ENCFF293RIK
CPSF7	ENCFF221ICX	ENCFF359NWF	ENCFF236XCF	ENCFF901WHO
CSTF2	ENCFF938DQM	ENCFF866AHO	ENCFF572NPL	ENCFF689YCR
CSTF2T	ENCFF334UJK	ENCFF529NFO	ENCFF057JNE	ENCFF294SKR
DAZAP1	ENCFF535DJP	ENCFF427XIT	ENCFF566IXO	ENCFF394YXV
DDX1	ENCFF727IBQ	ENCFF902WUX	ENCFF784SML	ENCFF487UNM
DDX19B	ENCFF022GNJ	ENCFF430RBN	ENCFF566XUG	ENCFF486GXG
DDX21	ENCFF026VLT	ENCFF332RXE	ENCFF057JNE	ENCFF294SKR

Continued on next page

Table S3 – *Continued from previous page*

Target	KD1	KD2	Ctrl1	Ctrl2
DDX24	ENCFF327HAD	ENCFF805KIS	ENCFF057JNE	ENCFF294SKR
DDX27	ENCFF414WJD	ENCFF210PWB	ENCFF572NPL	ENCFF689YCR
DDX28	ENCFF187ZFL	ENCFF875KOM	ENCFF572NPL	ENCFF689YCR
DDX3X	ENCFF572OTL	ENCFF196CGJ	ENCFF989TXQ	ENCFF558ZFO
DDX47	ENCFF487JHR	ENCFF161KIE	ENCFF357IOR	ENCFF239JNY
DDX5	ENCFF980OCO	ENCFF435DYK	ENCFF045COW	ENCFF668JLY
DDX52	ENCFF608NGH	ENCFF653INZ	ENCFF574DLW	ENCFF847SRU
DDX55	ENCFF876ECR	ENCFF193SMU	ENCFF572NPL	ENCFF689YCR
DDX59	ENCFF228NAK	ENCFF802SDT	ENCFF738YFT	ENCFF293RIK
DDX6	ENCFF365JNK	ENCFF725RWM	ENCFF989TXQ	ENCFF558ZFO
DHX30	ENCFF751TAL	ENCFF402UGA	ENCFF784SML	ENCFF487UNM
DKC1	ENCFF572VRD	ENCFF454QYL	ENCFF566XUG	ENCFF486GXG
DNAJC2	ENCFF534TBB	ENCFF878DOO	ENCFF784SML	ENCFF487UNM
DNAJC21	ENCFF998JKT	ENCFF168QYA	ENCFF784SML	ENCFF487UNM
EEF2	ENCFF832THG	ENCFF477MYS	ENCFF574DLW	ENCFF847SRU
EFTUD2	ENCFF039BLK	ENCFF149WKD	ENCFF574DLW	ENCFF847SRU
EIF2S1	ENCFF064LCJ	ENCFF776MXG	ENCFF989TXQ	ENCFF558ZFO
EIF2S2	ENCFF079OVB	ENCFF642MGZ	ENCFF236XCF	ENCFF901WHO
EIF3D	ENCFF713UFE	ENCFF708DON	ENCFF574DLW	ENCFF847SRU
EIF3G	ENCFF794TBV	ENCFF808FDG	ENCFF057JNE	ENCFF294SKR
EIF4A3	ENCFF588VRV	ENCFF160XLT	ENCFF989TXQ	ENCFF558ZFO
EIF4B	ENCFF400ARN	ENCFF002YLP	ENCFF989TXQ	ENCFF558ZFO
EIF4G1	ENCFF756IDB	ENCFF563CZX	ENCFF566IXO	ENCFF394YXV
EIF4G2	ENCFF131UOO	ENCFF028ZEH	ENCFF566IXO	ENCFF394YXV
ESF1	ENCFF919BHG	ENCFF843LUG	ENCFF236XCF	ENCFF901WHO
ETF1	ENCFF700QOG	ENCFF086UVA	ENCFF742NXL	ENCFF340UAV
EWSR1	ENCFF398AQF	ENCFF084OLA	ENCFF566XUG	ENCFF486GXG
EXOSC9	ENCFF793ZQF	ENCFF613CSR	ENCFF057JNE	ENCFF294SKR
FAM120A	ENCFF784QKI	ENCFF143FVG	ENCFF574DLW	ENCFF847SRU
FASTKD1	ENCFF393OPX	ENCFF648JHO	ENCFF869IKC	ENCFF996UTY
FASTKD2	ENCFF850TNI	ENCFF330THZ	ENCFF057JNE	ENCFF294SKR
FIP1L1	ENCFF127EMC	ENCFF253SZA	ENCFF236XCF	ENCFF901WHO
FKBP4	ENCFF982ALW	ENCFF257YRE	ENCFF572NPL	ENCFF689YCR

Continued on next page

Table S3 – *Continued from previous page*

Target	KD1	KD2	Ctrl1	Ctrl2
FMR1	ENCFF116MWT	ENCFF913XXV	ENCFF566IXO	ENCFF394YXV
FTO	ENCFF408FIB	ENCFF098ONQ	ENCFF738YFT	ENCFF293RIK
FUBP3	ENCFF513ADH	ENCFF880UOR	ENCFF926DGG	ENCFF718LIT
FXR1	ENCFF445CHH	ENCFF969RLT	ENCFF989TXQ	ENCFF558ZFO
G3BP1	ENCFF844ZHZ	ENCFF529THE	ENCFF989TXQ	ENCFF558ZFO
G3BP2	ENCFF478UOM	ENCFF739NFG	ENCFF989TXQ	ENCFF558ZFO
GEMIN5	ENCFF068KMA	ENCFF413TAT	ENCFF574DLW	ENCFF847SRU
GNB2L1	ENCFF070ISC	ENCFF506XFA	ENCFF261BLR	ENCFF966QBV
GPKOW	ENCFF861JDA	ENCFF603KNO	ENCFF738YFT	ENCFF293RIK
GRSF1	ENCFF016WDI	ENCFF506AFF	ENCFF869IKC	ENCFF996UTY
GRWD1	ENCFF420WYN	ENCFF181DMT	ENCFF261BLR	ENCFF966QBV
HDGF	ENCFF745GYC	ENCFF456MAS	ENCFF566XUG	ENCFF486GXG
HNRNPA0	ENCFF423AHV	ENCFF721QNS	ENCFF869IKC	ENCFF996UTY
HNRNPA1	ENCFF350IPK	ENCFF045OGG	ENCFF784SML	ENCFF487UNM
HNRNPA2B1	ENCFF606DES	ENCFF997UFK	ENCFF566IXO	ENCFF394YXV
HNRNPAB	ENCFF840CJZ	ENCFF651DBC	ENCFF869IKC	ENCFF996UTY
HNRNPC	ENCFF181LSI	ENCFF497PPO	ENCFF784SML	ENCFF487UNM
HNRNPD	ENCFF085OET	ENCFF830SPJ	ENCFF869IKC	ENCFF996UTY
HNRNPF	ENCFF438XHW	ENCFF593FFJ	ENCFF784SML	ENCFF487UNM
HNRNPK	ENCFF899ZBQ	ENCFF433BRP	ENCFF989TXQ	ENCFF558ZFO
HNRNPL	ENCFF536UGZ	ENCFF869LKX	ENCFF566IXO	ENCFF394YXV
HNRNPLL	ENCFF966FYM	ENCFF111KOR	ENCFF738YFT	ENCFF293RIK
HNRNPM	ENCFF601DUB	ENCFF240HMJ	ENCFF236XCF	ENCFF901WHO
HNRNPU	ENCFF292RTX	ENCFF516UIQ	ENCFF784SML	ENCFF487UNM
HNRNPUL1	ENCFF134YSM	ENCFF596UTY	ENCFF686VNL	ENCFF430GWF
HSPD1	ENCFF359GYZ	ENCFF471QSO	ENCFF093UUE	ENCFF870WUB
IGF2BP2	ENCFF817RTM	ENCFF534YDS	ENCFF989TXQ	ENCFF558ZFO
IGF2BP3	ENCFF753NXB	ENCFF441NPW	ENCFF989TXQ	ENCFF558ZFO
ILF2	ENCFF150WUU	ENCFF886MQA	ENCFF784SML	ENCFF487UNM
ILF3	ENCFF485RWH	ENCFF073SBH	ENCFF869IKC	ENCFF996UTY
KHDRBS1	ENCFF831SBV	ENCFF813BGP	ENCFF869IKC	ENCFF996UTY
KHSRP	ENCFF021RRG	ENCFF310LTU	ENCFF989TXQ	ENCFF558ZFO
KIF1C	ENCFF165QIV	ENCFF576IMD	ENCFF566XUG	ENCFF486GXG

Continued on next page

Table S3 – *Continued from previous page*

Target	KD1	KD2	Ctrl1	Ctrl2
KRR1	ENCFF497UDR	ENCFF576VLK	ENCFF093UUE	ENCFF870WUB
LARP4	ENCFF890GSN	ENCFF760RVL	ENCFF930OYV	ENCFF934UWJ
LARP7	ENCFF870DWP	ENCFF787GCZ	ENCFF261BLR	ENCFF966QBV
LIN28B	ENCFF511GIN	ENCFF446IUZ	ENCFF261BLR	ENCFF966QBV
LSM11	ENCFF502SMN	ENCFF939XZO	ENCFF261BLR	ENCFF966QBV
MAGOH	ENCFF950HSR	ENCFF045JYP	ENCFF989TXQ	ENCFF558ZFO
MARK2	ENCFF294MIQ	ENCFF692GMY	ENCFF261BLR	ENCFF966QBV
MATR3	ENCFF780SYO	ENCFF246QJB	ENCFF989TXQ	ENCFF558ZFO
METAP2	ENCFF619KWP	ENCFF249ZQO	ENCFF574DLW	ENCFF847SRU
MSI2	ENCFF306PRN	ENCFF915LIM	ENCFF236XCF	ENCFF901WHO
MTPAP	ENCFF699CXV	ENCFF943JPB	ENCFF738YFT	ENCFF293RIK
NAA15	ENCFF182PZY	ENCFF594GKW	ENCFF236XCF	ENCFF901WHO
NCBP2	ENCFF594LYM	ENCFF083LTV	ENCFF989TXQ	ENCFF558ZFO
NELFE	ENCFF091TXV	ENCFF401AVH	ENCFF261RLR	ENCFF834TYC
NIP7	ENCFF354LYI	ENCFF774DZT	ENCFF261BLR	ENCFF966QBV
NKRF	ENCFF294MHK	ENCFF100KDW	ENCFF093UUE	ENCFF870WUB
NOL12	ENCFF806ZKL	ENCFF466PAF	ENCFF566IXO	ENCFF394YXV
NONO	ENCFF966TUI	ENCFF902KNB	ENCFF572NPL	ENCFF689YCR
NPM1	ENCFF373WYF	ENCFF731QCQ	ENCFF686VNL	ENCFF430GWF
NSUN2	ENCFF322GMR	ENCFF269GWO	ENCFF738YFT	ENCFF293RIK
NUFIP2	ENCFF306PZW	ENCFF152DFW	ENCFF686VNL	ENCFF430GWF
NUP35	ENCFF066VPP	ENCFF261DTU	ENCFF021BCS	ENCFF762FFH
NUSAP1	ENCFF116WUD	ENCFF410NIR	ENCFF261BLR	ENCFF966QBV
PA2G4	ENCFF687LTL	ENCFF136SOB	ENCFF574DLW	ENCFF847SRU
PABPC1	ENCFF927GPT	ENCFF698UKF	ENCFF869IKC	ENCFF996UTY
PABPC4	ENCFF359BAT	ENCFF560RJQ	ENCFF989TXQ	ENCFF558ZFO
PARN	ENCFF558REQ	ENCFF038YUS	ENCFF784SML	ENCFF487UNM
PCBP1	ENCFF463AMS	ENCFF174ZQQ	ENCFF261RLR	ENCFF834TYC
PES1	ENCFF333LCM	ENCFF964PAH	ENCFF566XUG	ENCFF486GXG
PHF6	ENCFF858EYL	ENCFF306MQU	ENCFF261BLR	ENCFF966QBV
PKM	ENCFF249PUC	ENCFF474EDD	ENCFF574DLW	ENCFF847SRU
PNPT1	ENCFF875SZJ	ENCFF713XBL	ENCFF869IKC	ENCFF996UTY
PPIG	ENCFF374SOY	ENCFF585WPZ	ENCFF093UUE	ENCFF870WUB

Continued on next page

Table S3 – *Continued from previous page*

Target	KD1	KD2	Ctrl1	Ctrl2
PPIL4	ENCFF069NTO	ENCFF537PSL	ENCFF784SML	ENCFF487UNM
PRPF8	ENCFF727IHN	ENCFF192LSC	ENCFF572NPL	ENCFF689YCR
PSIP1	ENCFF048MHS	ENCFF412QTU	ENCFF261BLR	ENCFF966QBV
PTBP1	ENCFF145TKU	ENCFF855TDM	ENCFF261RLR	ENCFF834TYC
PUF60	ENCFF937YLH	ENCFF064JDU	ENCFF566IXO	ENCFF394YXV
PUM1	ENCFF300LBV	ENCFF261TUX	ENCFF566IXO	ENCFF394YXV
PUM2	ENCFF239VRF	ENCFF464WLH	ENCFF566IXO	ENCFF394YXV
PUS1	ENCFF727ATR	ENCFF317YKN	ENCFF566XUG	ENCFF486GXG
QKI	ENCFF354FHT	ENCFF819VYL	ENCFF261RLR	ENCFF834TYC
RBFOX2	ENCFF880HKN	ENCFF511KFD	ENCFF686VNL	ENCFF430GWF
RBM15	ENCFF276WQL	ENCFF057WEZ	ENCFF278EWB	ENCFF376SXK
RBM17	ENCFF993AIW	ENCFF442JOE	ENCFF686VNL	ENCFF430GWF
RBM22	ENCFF030OLZ	ENCFF369NEZ	ENCFF742NXL	ENCFF340UAV
RBM25	ENCFF518EYV	ENCFF322NIO	ENCFF261RLR	ENCFF834TYC
RBM27	ENCFF541ADT	ENCFF241FYW	ENCFF261BLR	ENCFF966QBV
RBM39	ENCFF343OAG	ENCFF942AVR	ENCFF686VNL	ENCFF430GWF
RBM47	ENCFF680QZV	ENCFF597HYM	ENCFF869IKC	ENCFF996UTY
RCC2	ENCFF267UFO	ENCFF706HMR	ENCFF261BLR	ENCFF966QBV
RECQL	ENCFF609EEV	ENCFF867UWM	ENCFF261RLR	ENCFF834TYC
RPL23A	ENCFF916TOV	ENCFF059YGS	ENCFF261BLR	ENCFF966QBV
RPLP0	ENCFF380JZX	ENCFF781BVK	ENCFF261BLR	ENCFF966QBV
RPS19	ENCFF707VTW	ENCFF366TRL	ENCFF574DLW	ENCFF847SRU
RPS2	ENCFF108BIO	ENCFF259TDR	ENCFF261BLR	ENCFF966QBV
RPS3A	ENCFF054JEN	ENCFF392VDF	ENCFF869IKC	ENCFF996UTY
RPS5	ENCFF455IHZ	ENCFF583EQH	ENCFF261BLR	ENCFF966QBV
RRP9	ENCFF005NNX	ENCFF778BWF	ENCFF572NPL	ENCFF689YCR
SAFB2	ENCFF117ZFN	ENCFF403DFM	ENCFF784SML	ENCFF487UNM
SART3	ENCFF056ATS	ENCFF254RRV	ENCFF869IKC	ENCFF996UTY
SBDS	ENCFF801BRP	ENCFF642WPV	ENCFF869IKC	ENCFF996UTY
SERBP1	ENCFF674ZQN	ENCFF171XGJ	ENCFF574DLW	ENCFF847SRU
SF1	ENCFF586WHY	ENCFF772YRQ	ENCFF686VNL	ENCFF430GWF
SF3A3	ENCFF923LZN	ENCFF677KRY	ENCFF168UDP	ENCFF321ZFN
SF3B1	ENCFF349YJR	ENCFF465NDY	ENCFF738YFT	ENCFF293RIK

Continued on next page

Table S3 – *Continued from previous page*

Target	KD1	KD2	Ctrl1	Ctrl2
SF3B4	ENCFF301YOD	ENCFF784ZDW	ENCFF261RLR	ENCFF834TYC
SFPQ	ENCFF611BJW	ENCFF402HCN	ENCFF686VNL	ENCFF430GWF
SLBP	ENCFF636XLW	ENCFF956LAK	ENCFF566IXO	ENCFF394YXV
SMN1	ENCFF489WVZ	ENCFF576QGY	ENCFF784SML	ENCFF487UNM
SNRNP200	ENCFF132QOS	ENCFF809FCF	ENCFF686VNL	ENCFF430GWF
SNRNP70	ENCFF186MWG	ENCFF305IBD	ENCFF738YFT	ENCFF293RIK
SRFBP1	ENCFF041MUH	ENCFF535THG	ENCFF742NXL	ENCFF340UAV
SRP68	ENCFF137KFS	ENCFF187GDS	ENCFF572NPL	ENCFF689YCR
SRSF1	ENCFF856VSZ	ENCFF942DHD	ENCFF261RLR	ENCFF834TYC
SRSF3	ENCFF754ESC	ENCFF365PLL	ENCFF738YFT	ENCFF293RIK
SRSF5	ENCFF332XZD	ENCFF154XVC	ENCFF566IXO	ENCFF394YXV
SRSF7	ENCFF642TXU	ENCFF222WYC	ENCFF261RLR	ENCFF834TYC
SRSF9	ENCFF754SUW	ENCFF664KDY	ENCFF261RLR	ENCFF834TYC
SSB	ENCFF647DVY	ENCFF105XIV	ENCFF742NXL	ENCFF340UAV
SSRP1	ENCFF173IMV	ENCFF814EUR	ENCFF093UUE	ENCFF870WUB
STAU1	ENCFF423PGY	ENCFF666GBG	ENCFF236XCF	ENCFF901WHO
STIP1	ENCFF608WDW	ENCFF504JGG	ENCFF869IKC	ENCFF996UTY
SUCLG1	ENCFF704JGS	ENCFF250DLK	ENCFF566IXO	ENCFF394YXV
SUGP2	ENCFF434QDG	ENCFF622PET	ENCFF093UUE	ENCFF870WUB
SUPT6H	ENCFF179AAM	ENCFF387ASY	ENCFF236XCF	ENCFF901WHO
SUPV3L1	ENCFF082CNV	ENCFF734OAK	ENCFF572NPL	ENCFF689YCR
TAF15	ENCFF777VEO	ENCFF531USK	ENCFF261RLR	ENCFF834TYC
TARDBP	ENCFF759ZRY	ENCFF835ZPC	ENCFF738YFT	ENCFF293RIK
TBRG4	ENCFF328RQK	ENCFF496DOM	ENCFF093UUE	ENCFF870WUB
TFIP11	ENCFF683CRU	ENCFF319TPH	ENCFF572NPL	ENCFF689YCR
TIA1	ENCFF298LYN	ENCFF243YSW	ENCFF261RLR	ENCFF834TYC
TIAL1	ENCFF593CFB	ENCFF273JPU	ENCFF784SML	ENCFF487UNM
TRIM56	ENCFF359RCT	ENCFF816AOJ	ENCFF784SML	ENCFF487UNM
TROVE2	ENCFF119ZCG	ENCFF115CEE	ENCFF574DLW	ENCFF847SRU
TUFM	ENCFF328WOU	ENCFF709PZI	ENCFF566IXO	ENCFF394YXV
U2AF1	ENCFF163IXV	ENCFF172LGC	ENCFF236XCF	ENCFF901WHO
U2AF2	ENCFF911EJE	ENCFF821FZB	ENCFF686VNL	ENCFF430GWF
UBE2L3	ENCFF022GVT	ENCFF416BYL	ENCFF566IXO	ENCFF394YXV

Continued on next page

Table S3 – *Continued from previous page*

Target	KD1	KD2	Ctrl1	Ctrl2
UCHL5	ENCFF432SEN	ENCFF300CDS	ENCFF572NPL	ENCFF689YCR
UPF1	ENCFF850ORL	ENCFF819BTX	ENCFF168UDP	ENCFF321ZFN
UPF2	ENCFF023KBS	ENCFF737IEW	ENCFF572NPL	ENCFF689YCR
UTP18	ENCFF977IXL	ENCFF202TGW	ENCFF168UDP	ENCFF321ZFN
UTP3	ENCFF131RNR	ENCFF876DBS	ENCFF168UDP	ENCFF321ZFN
XPO5	ENCFF386NUM	ENCFF234HPE	ENCFF869IKC	ENCFF996UTY
XRN2	ENCFF450HWR	ENCFF789QEA	ENCFF572NPL	ENCFF689YCR
YBX3	ENCFF296FTT	ENCFF036XRE	ENCFF784SML	ENCFF487UNM
ZNF622	ENCFF846YSQ	ENCFF776IMC	ENCFF168UDP	ENCFF321ZFN

Table S3: The list of accession numbers (*24, 25*) of RBP shRNA-knockdown followed by RNA-seq. Two knock-down bioreplicates (KD1, KD2) and their control experiments (Ctrl1, Ctrl2).

Target	eCLIP1	eCLIP2
AGGF1	ENCFF388PDG	ENCFF283MCJ
AKAP1	ENCFF271UXH	ENCFF807KPM
BCCIP	ENCFF627YDB	ENCFF698TRT
BCLAF1	ENCFF691JPV	ENCFF858HBB
BUD13	ENCFF364QPG	ENCFF929ITC
CDC40	ENCFF890AXT	ENCFF513MLD
CSTF2	ENCFF892JUQ	ENCFF370ZBJ
CSTF2T	ENCFF700HIR	ENCFF786JAM
DDX3X	ENCFF798XQM	ENCFF799ICU
DDX52	ENCFF591WOA	ENCFF539JUN
DDX55	ENCFF315AVG	ENCFF584QHX
DDX59	ENCFF441KPC	ENCFF485MSN
DDX6	ENCFF438KSN	ENCFF650BQD
DGCR8	ENCFF674MHQ	ENCFF382SXP
DHX30	ENCFF506NPG	ENCFF191HRQ
DKC1	ENCFF074ATJ	ENCFF043XKO
EFTUD2	ENCFF511ZTQ	ENCFF955RUR

Continued on next page

Table S4 – *Continued from previous page*

Target	eCLIP1	eCLIP2
EIF3D	ENCFF650RVF	ENCFF738CPK
EIF3H	ENCFF770FPA	ENCFF582LGR
FAM120A	ENCFF511SXK	ENCFF287ADK
FASTKD2	ENCFF503DBN	ENCFF136QLO
FKBP4	ENCFF993XGD	ENCFF585PKG
FTO	ENCFF655JNU	ENCFF925SGC
FUBP3	ENCFF410ITK	ENCFF714TFP
G3BP1	ENCFF934JFZ	ENCFF165JYX
GRSF1	ENCFF733VOV	ENCFF451GQU
GRWD1	ENCFF689GDA	ENCFF735YNV
GTF2F1	ENCFF092IJR	ENCFF240QYG
HNRNPA1	ENCFF535MAE	ENCFF604WJZ
HNRNPC	ENCFF401SPQ	ENCFF738FJS
HNRNPK	ENCFF877KBJ	ENCFF556EBT
HNRNPL	ENCFF581SSQ	ENCFF415HDB
HNRNPM	ENCFF542FRX	ENCFF040JSC
HNRNPU	ENCFF024FCB	ENCFF587FNW
HNRNPUL1	ENCFF676GXG	ENCFF801KHR
IGF2BP1	ENCFF976DBP	ENCFF486BXN
IGF2BP3	ENCFF858ZLX	ENCFF175PMW
ILF3	ENCFF460MRE	ENCFF535COB
KHSRP	ENCFF509GWT	ENCFF891IAC
LARP4	ENCFF288SGB	ENCFF130RAQ
LARP7	ENCFF699ZLU	ENCFF440FLF
LIN28B	ENCFF976KYV	ENCFF381VVY
LSM11	ENCFF870HZF	ENCFF220ECM
MATR3	ENCFF874BAL	ENCFF333CUY
NCBP2	ENCFF047YUZ	ENCFF500HBQ
NIP7	ENCFF077RVD	ENCFF527MFS
NKRF	ENCFF233SNH	ENCFF864UGQ
NOL12	ENCFF716SCR	ENCFF515WRY
PCBP1	ENCFF252BJN	ENCFF940CJE
PCBP2	ENCFF074AQN	ENCFF322DXB

Continued on next page

Table S4 – *Continued from previous page*

Target	eCLIP1	eCLIP2
PPIG	ENCFF597JSM	ENCFF603DSB
PRPF8	ENCFF646XFI	ENCFF156PFJ
PTBP1	ENCFF154BAE	ENCFF471GJE
QKI	ENCFF525PZA	ENCFF459ANM
RBFOX2	ENCFF639MYI	ENCFF664WCU
RBM15	ENCFF017HYK	ENCFF971BBY
RBM22	ENCFF353XWS	ENCFF990UNN
RBM5	ENCFF980CBO	ENCFF663GBN
RPS3	ENCFF843WBE	ENCFF163XOB
SF3A3	ENCFF642VTG	ENCFF075FPZ
SF3B4	ENCFF581RVY	ENCFF363PGR
SFPQ	ENCFF221WOF	ENCFF919FIY
SLTM	ENCFF870XZV	ENCFF892IWC
SMNDC1	ENCFF230AER	ENCFF240MCZ
SND1	ENCFF054ZXE	ENCFF749TYO
SRSF1	ENCFF179SCM	ENCFF184TBM
SRSF7	ENCFF774EDY	ENCFF913UPA
SRSF9	ENCFF125UVA	ENCFF409GPI
SSB	ENCFF828WCR	ENCFF746RLH
SUB1	ENCFF033IXH	ENCFF978MMK
SUGP2	ENCFF187DUQ	ENCFF699DTX
SUPV3L1	ENCFF882KLS	ENCFF089NRQ
TAF15	ENCFF603JTU	ENCFF063BTH
TBRG4	ENCFF472CCD	ENCFF454ZHL
TIA1	ENCFF055IIM	ENCFF358SGV
TIAL1	ENCFF456DAU	ENCFF473IIG
TRA2A	ENCFF210QRC	ENCFF955HGE
TROVE2	ENCFF515SVK	ENCFF029CYV
U2AF1	ENCFF078SCZ	ENCFF295GHA
U2AF2	ENCFF444TCK	ENCFF523JWI
UCHL5	ENCFF305LRB	ENCFF171KTG
UPF1	ENCFF848JHB	ENCFF483STL
UTP18	ENCFF227SZN	ENCFF235ASG

Continued on next page

Table S4 – *Continued from previous page*

Target	eCLIP1	eCLIP2
XPO5	ENCFF756YND	ENCFF028MVM
XRCC6	ENCFF225IXQ	ENCFF381LFD
XRN2	ENCFF845EBP	ENCFF944KMN
YBX3	ENCFF268SII	ENCFF904UKX

Table S4: The list of accession numbers (*24, 25*) of RBP eCLIP peaks; two bioreplicates (eCLIP1 and eCLIP2).

Supplementary References

1. Pervouchine, D. D. IRBIS: a systematic search for conserved complementarity. *RNA* **20**, 1519–1531 (2014).
2. Smith, T. F. & Waterman, M. S. Identification of common molecular subsequences. *J. Mol. Biol.* **147**, 195–197 (1981).
3. Lorenz, R. *et al.* ViennaRNA Package 2.0. *Algorithms Mol Biol* **6**, 26 (2011).
4. Mathews, D. H., Sabina, J., Zuker, M. & Turner, D. H. Expanded sequence dependence of thermodynamic parameters improves prediction of RNA secondary structure. *J. Mol. Biol.* **288**, 911–940 (1999).
5. Singh, N. N., Androphy, E. J. & Singh, R. N. An extended inhibitory context causes skipping of exon 7 of SMN2 in spinal muscular atrophy. *Biochem Biophys Res Commun* **315**, 381–388 (2004).
6. Miyaso, H. *et al.* An intronic splicing enhancer element in survival motor neuron (SMN) pre-mRNA. *J Biol Chem* **278**, 15825–15831 (2003).
7. Singh, N. N. *et al.* An intronic structure enabled by a long-distance interaction serves as a novel target for splicing correction in spinal muscular atrophy. *Nucleic Acids Res.* **41**, 8144–8165 (2013).
8. Singh, N. N., Lee, B. M. & Singh, R. N. Splicing regulation in spinal muscular atrophy by an RNA structure formed by long-distance interactions. *Ann. N. Y. Acad. Sci.* **1341**, 176–187 (2015).
9. Wong, M. S., Shay, J. W. & Wright, W. E. Regulation of human telomerase splicing by RNA:RNA pairing. *Nat Commun* **5**, 3306 (2014).
10. Lin, Y., Schmidt, B. F., Bruchez, M. P. & McManus, C. J. Structural analyses of NEAT1 lncRNAs suggest long-range RNA interactions that may contribute to paraspeckle architecture. *Nucleic Acids Res* **46**, 3742–3752 (2018).
11. Varani, L. *et al.* Structure of tau exon 10 splicing regulatory element RNA and destabilization by mutations of frontotemporal dementia and parkinsonism linked to chromosome 17. *Proc Natl Acad Sci U S A* **96**, 8229–8234 (1999).
12. Jiang, Z., Cote, J., Kwon, J. M., Goate, A. M. & Wu, J. Y. Aberrant splicing of tau pre-mRNA caused by intronic mutations associated with the inherited dementia frontotemporal dementia with parkinsonism linked to chromosome 17. *Mol Cell Biol* **20**, 4036–4048 (2000).
13. D'Souza, I. *et al.* Missense and silent tau gene mutations cause frontotemporal dementia with parkinsonism-chromosome 17 type, by affecting multiple alternative RNA splicing regulatory elements. *Proc Natl Acad Sci U S A* **96**, 5598–5603 (1999).

14. Grover, A. *et al.* 5' splice site mutations in tau associated with the inherited dementia FTDP-17 affect a stem-loop structure that regulates alternative splicing of exon 10. *J Biol Chem* **274**, 15134–15143 (1999).

15. Baraniak, A. P., Lasda, E. L., Wagner, E. J. & Garcia-Blanco, M. A. A stem structure in fibroblast growth factor receptor 2 transcripts mediates cell-type-specific splicing by approximating intronic control elements. *Mol Cell Biol* **23**, 9327–9337 (2003).

16. Warf, M. B. & Berglund, J. A. MBNL binds similar RNA structures in the CUG repeats of myotonic dystrophy and its pre-mRNA substrate cardiac troponin T. *RNA* **13**, 2238–2251 (2007).

17. Luscieti, S. *et al.* Novel mutations in the ferritin-L iron-responsive element that only mildly impair IRP binding cause hereditary hyperferritinaemia cataract syndrome. *Orphanet J Rare Dis* **8**, 30 (2013).

18. Bartoszewski, R. A. *et al.* A synonymous single nucleotide polymorphism in DeltaF508 CFTR alters the secondary structure of the mRNA and the expression of the mutant protein. *J Biol Chem* **285**, 28741–28748 (2010).

19. Estes, P. A., Cooke, N. E. & Liebhaber, S. A. A native RNA secondary structure controls alternative splice-site selection and generates two human growth hormone isoforms. *J. Biol. Chem.* **267**, 14902–14908 (1992).

20. Higashide, S. *et al.* Identification of regulatory cis-acting elements for alternative splicing of presenilin 2 exon 5 under hypoxic stress conditions. *J Neurochem* **91**, 1191–1198 (2004).

21. Muro, A. F. *et al.* Regulation of fibronectin EDA exon alternative splicing: possible role of RNA secondary structure for enhancer display. *Mol Cell Biol* **19**, 2657–2671 (1999).

22. McAlinden, A., Havlioglu, N., Liang, L., Davies, S. R. & Sandell, L. J. Alternative splicing of type II procollagen exon 2 is regulated by the combination of a weak 5' splice site and an adjacent intronic stem-loop cis element. *J Biol Chem* **280**, 32700–32711 (2005).

23. Kalinina, M. *et al.* Multiple competing RNA structures dynamically control alternative splicing in the human ATE1 gene. *Nucleic Acids Res* **49**, 479–490 (2021).

24. Dunham, I. *et al.* An integrated encyclopedia of DNA elements in the human genome. *Nature* **489**, 57–74 (2012).

25. Sloan, C. A. *et al.* ENCODE data at the ENCODE portal. *Nucleic Acids Res.* **44**, D726–732 (2016).
