# Supplementary material for: Conserved long-range base pairings are associated with pre-mRNA processing of human genes
Source: Nat Commun. 2021 Apr 16;12:2300. doi: 10.1038/s41467-021-22549-7 (PMC8052449; doi:10.1038/s41467-021-22549-7)
Supplement: Supplementary file 7 — Description of Additional Supplementary Files [file 41467_2021_22549_MOESM7_ESM.docx]

Description of additional supplementary files

Title: SupplementaryDataFile 1

Description: The full list of PCCRs, GRCh37 Human Genome assembly.

Title: SupplementaryDataFile 2

Description: The full list of PCCRs, GRCh38 Human Genome assembly. Title: SupplementaryDataFile 3: RNA bridges, GRCh37 Human Genome assembly.

Title: SupplementaryDataFile 4

Description: Exon loop-outs, GRCh37 Human Genome assembly.

Title: SupplementaryDataFile 5

Description: A stringent set of intramolecular RIC-seq RNA contacts, provided as a courtesy of Prof. Xue (26
